# Identification and Characterization of miRNAs in Response to *Leishmania donovani* Infection: Delineation of Their Roles in Macrophage Dysfunction

**DOI:** 10.3389/fmicb.2017.00314

**Published:** 2017-03-02

**Authors:** Neeraj Tiwari, Vinod Kumar, Mallikarjuna Rao Gedda, Ashish K. Singh, Vijay K. Singh, Surya P. Singh, Rakesh K. Singh

**Affiliations:** ^1^Molecular Immunology Group, Department of Biochemistry, Institute of Science, Banaras Hindu UniversityVaranasi, India; ^2^Department of Parasitology and Molecular Biology, Rajendra Memorial Research InstitutePatna, India; ^3^Bioinformatics Programme, Centre for Biological Science, Central University of South BiharPatna, India; ^4^Division of Emerging and Transfusion Transmitted Diseases, Center for Biologics Evaluation and Research, Food and Drug AdministrationSilver Spring, MD, USA

**Keywords:** *L*. *donovani*, miRNA, biomarkers, macrophages, dysfunction

## Abstract

The outcome of *Leishmania* infection depends on parasite abilities to evade host immune response and its survival in hostile environment of host macrophages. Despite a wealth of gained crucial information, parasite strategies by which it dampens host macrophage functions remain poorly understood. Micro RNAs (miRNAs) are evolutionarily conserved class of endogenous 22-nucleotide small non-coding RNA gene products, described to participate in the regulation of almost every cellular process investigated so far. In this study, we identified 940 miRNAs in *Leishmania donovani* infected macrophages by *de novo* sequencing out of which levels of 85 miRNAs were found to be consistently modified by parasite infection. Herein, we report the functional characteristics of 10 miRNAs i.e., mir-3620, mir-6385, mir-6973a, mir-6996, mir-328, mir-8113, mir-3473f, mir-763, mir-6540, and mir-1264 that were differentially but constantly regulated in infected macrophages for their role in regulation of macrophage effector functions. The target gene prediction and biological interaction analysis revealed involvement of these miRNAs in various biological processes such as apoptosis inhibition, phagocytosis, drug response, and T cell phenotypic transitions. These findings could contribute for the better understanding of macrophages dysfunction and leishmanial pathogenesis. Further, the identified miRNAs could also be used as biomarker/s in diagnosis, prognosis, and therapeutics of *Leishmania* infection.

## Introduction

Leishmaniasis is considered as neglected tropical disease however, at present it is prevalent in more than 98 countries and associated with significant mortality and morbidity (Alvar et al., 2012). Out of 53 described species of *Leishmania* parasites, 20 are known to cause human pathogenesis (Akhoundi et al., 2016). All *Leishmania* species exhibit digenetic life cycle; first, the flagellated extracellular infective promastigote form that resides in the gut of sand fly; and second, a non-flagellated amastigote form, which resides within phagolysosomal compartment of host macrophages (Kamhawi, 2006). Based on its clinical manifestations, the disease is classified into three types: cutaneous (CL), mucocutaneous (MCL), and visceral (VL) leishmaniasis, which differ in their immunopathologies, degree of morbidity and mortality.

Approximately 350 million people are at risk worldwide by all three forms with 12 million cases of infection (Akhoundi et al., 2016). In disease endemic countries, the estimated annual incidence of CL is ~0.7–1.3 million cases and 0.2–0.4 million cases of VL (WHO, 2016). More than 90% of VL cases come from six countries i.e., Brazil, Ethiopia, India, Somalia, South Sudan, and Sudan (WHO, 2016). VL is caused by *Leishmania donovani* in Indian subcontinent, by *Leishmania infantum* in North Africa and Southern Europe and by *Leishmania chagasi* in Latin American countries. Out of three pathogenic states, VL infections are fatal, if left untreated and responsible for ~40,000 deaths per year, worldwide (Ready, 2014).

The chemotherapeutics measures to control leishmanial infections are very limited and lack of a vaccine, either prophylactic or preventive, further complicates this issue. The pentavalent antimonial compounds, considered only true antileishmanial, have been the mainstay to treat VL soon after identification of *Leishmania* parasites. Although, antimonials are in use till date but parasites resistance to these compounds are being reported at an alarming rate especially in Indian subcontinent (Chakravarty and Sundar, 2010). In lieu of an alternative antileishmanial drug, leishmanial infections are being treated by either antimicrobial such as pentamidine (originally developed in search of a hypoglycemic agent and were used in treatment of African trypanosomiasis in late 1930s), anti-fungal (amphotericin B, used in early 1960s to treat leishmaniasis), or antitumor (miltefosine; 1998) drugs, which are also associated with serious side effects (Jha, 1983; Croft et al., 1987; Mishra et al., 1992; Fouce et al., 1998; Sundar et al., 1998). Later, in the year 1999 a liposomal formulation of amphotericin B (ambisome) was approved by Food and Drug Administration (Meyerhoff, 1999). Out of these three drugs, only ambisome and miltefosine are currently being used in disease endemic regions. These drugs never produce sterile cure and as a consequence few individuals develop post kala-azar dermal leishmaniasis (PKDL) even after successful cure, which again results in serious morbidity and mortality (Mukhopadhyay et al., 2014). In addition, resistance has also been observed against these two drugs in clinical isolates (Purkait et al., 2012; Mishra and Singh, 2013). Therefore, in lieu of unavailability of a vaccine and a serious threat for development of drug resistant parasites against current drug regimen, the search for alternate control strategies are highly needed to counter leishmanial infections.

*Leishmania*, a protozoan intracellular parasite, resides and proliferate in hostile environment of host macrophages, by smartly silencing their effector properties, which is characterized by decreased production of reactive oxygen species (ROS), nitric oxide (NOx), and pro-inflammatory cytokines such as TNF-α and IL-12 (Kima, 2007). In addition, the parasite also alters adaptive immune response and activates anti-inflammatory Th2 type T cells that suppress proliferation and functions of Th1 type T cells (Gannavaram et al., 2016). Indeed, all these events favor *Leishmania* persistence through inhibition of inflammatory cytokines production, poor antigen presentation and altered cellular signaling pathways in the favor of parasite survival (Chakraborty et al., 2005). The host resistance in leishmaniasis is related to effective clearance of parasites by macrophages and establishment of Th1 type immunity whereas an active IL-10 producing Th2 response is linked to disease susceptibility (Gupta et al., 2013; Ganguli et al., 2015).

Notwithstanding few host and parasitic factors responsible in suppression of macrophage effector properties are known but the exact mechanisms of macrophage dysfunction are largely unknown. MicroRNAs (miRNAs) are a subset of short non-coding RNAs, ~22 nucleotide (nt) long sequences that constitute an evolutionarily conserved system, associated with the regulation of biological and molecular functions at the post-transcriptional level by base pairing with target mRNAs (Bartel, 2004). They regulate the expression of target genes at the levels of mRNA stability and translation and help in survival of both, intra and extra cellular parasites (Bartel, 2009; Lu and Rothenberg, 2013; Baroni and Arrigo, 2014).

The role of various miRNAs such as miRNA-210 in *Leishmania major*, miRNA-29c in tuberculosis, miRNA-16 in malaria, miRNA-181 in *Helicobacter pylori* infection, miRNA-150 and miRNA-146b-5p in human immunodeficiency virus (HIV) have been found to be associated with either resistance or susceptibility (Wang et al., 2008; Lemaire et al., 2013; Verma et al., 2016). However, the way miRNAs regulate host macrophage effector functions in *L. donovani* infection remains to be elucidated. In this study, we identified 940 miRNAs of host macrophages by *de novo* sequencing and analyzed the functions of 10 miRNAs, which were differentially regulated for their regulatory roles in macrophage functions. Using defined experimental approaches i.e., expression profiling, gene ontology, and sequencing, this study provides evidence that the *L. donovani* strongly induces macrophage miRNAs that eventually down regulate its effector properties.

## Materials and methods:

### *L. donovani* promastigotes and macrophage culture

*L. donovani* (AG83) parasites were used in this study. The parasites virulence was maintained in BALB/c mice through serial passage. The motile promastigote forms of parasite were cultured in complete Dulbecco's Modified Eagle Medium (pH 7.2) (DMEM, Gibco, USA) containing 10% heat-inactivated fetal bovine serum (FBS, Gibco, USA), 2 mM L-glutamine, sodium bicarbonate, and antibiotics (Sigma Chemicals, USA); penicillin (100 U/ml), streptomycin (100 μg/ml), gentamycin (20 μg/ml) at 26°C in a BOD incubator. RAW 264.7 mice macrophage cell line procured from NCCS Pune, was maintained in DMEM medium supplemented with 10% FBS at 37°C in a humidified mixture of 5% CO_2_ atmosphere for further studies.

### Parasite infection and RNA isolation

Macrophages were infected with parasites for 6 h. After incubation, cells were thoroughly washed to remove non-internalized parasites and further cultured in fresh DMEM with 10% FBS and antibiotics in CO_2_ incubator at 37°C supplemented with 5% CO_2_. The cells without infection were used as control. After 24 h incubation post infection, total RNA was extracted using Tri® reagent (Sigma Chemicals, USA) following manufacturer's instructions. Briefly, cells were collected and pelleted by centrifuged at 500 g at 4°C for 15 min. The supernatant was removed and the cells were washed with PBS to remove complete media. The cells were then lysed in 300 μl of Tri® reagent and 120 μl chloroform. The suspension was centrifuged at 8,000 g at 25°C for 10 min. The upper aqueous layer was recovered and twice amount of isopropanol was added. The mixture was centrifuged again at 8,000 g at 4°C for 10 min and RNA pellets were collected. Finally, RNA pellets were washed three times with 70% DEPC ethanol to remove the impurities. Total RNA was first digested with RNAse free DNase (Fermantas, Germany) to avoid DNA contamination before use. Isolated RNAs were immediately preserved at −80°C.

### Small RNA library validation and next-generation sequencing

Small RNA Library was prepared using TrueSeq small RNA library prep kit (Illumina San Diego CA, USA) according to the manufacturer's instruction. This whole procedure requires adapter ligation, reverse transcription, PCR amplification, and pooled gel purification to generate a library product. For targeting miRNAs having a 3′ hydroxyl group resulting from enzymatic cleavage by Dicer or other RNA processing enzymes, RNA 3′ adapter was specifically modified to target miRNAs. Further, Adapters were ligated to each end of the RNAs and subsequently reverse transcribed to create single-stranded cDNA and sequenced using miRNA sequencing on an IlluminaHiSeq 2000 platform (Illumina, San Diego, CA, USA). By using a DNA specific chip 1 μl of the resuspended library construct was loaded on Agilent Technologies 2100 Bioanalyzer chip for small RNA Library validation. Prior to the sequencing, all the individual libraries were clustered together using TrueSeq Cluster kit V3-cBot-HS (HiSeq) in a single lane on an IlluminaHiSeq 2000 platform that generated 0–100 bp paired end reads.

### Data extraction and analysis; raw data filtering and miRBase V19 mapping

The raw data generated earlier in FastQ format was further filtered for high quality (>Q20 bases) reads of which low quality reads were removed and low quality bases were trimmed. Following adaptor sequences trimming, reads <18 bases were discarded as too short. Further, filtered high quality raw data was mapped on to miRBase V19. Same-strand matches of sRNAs to the miRBase databases were reported. The 0–3 mismatches were allowed for the search against miRBase. During this following reports were produced; miRNAs hits in an excel sheet along with matching coordinates in the genome and the precursor sequence. The miRNAs were matched with different variants of the miRNAs, such as: different mature sequences that can arise from the same precursor, annotated in miRBase as −5p, −s, or −as in the ID of the miRNA. Different precursors producing the same mature sequence, annotated as −1, −2, etc., in miRBase.

### Differential miRNA expression

The Excel sheet provided contained information about total and non-redundant (unique) sequence counts for each sample. Data obtained for each sample after the final filtering step was used for normalization. The normalized counts were given in “matching reads per 1 million total reads” to make them comparable between samples.

### Genome mapping and miRNA prediction criteria

The filtered sequences were further mapped to a genome of interest using PATMAN software with each mapping locus sequences with abundance of 5 or more being tested as miRNAs. Small RNAs read between 18 and 25 nt were considered as potential miRNA candidates and were tested as miRNAs. Along with this the length of sequences with 16 or fewer genomic matches were also tested as miRNAs. After the sequences mapped to the input genome, the clusters of sequences matching certain criteria were looked up by the software. Once a list of clusters was produced they were further analyzed in order to find likely miRNA candidates. The most abundant small RNA read within a cluster was chosen as the likely miRNA. Flanking sequences surrounding small RNA was extracted from the genome using a variety of window lengths. Each sequence window was then folded using RNA fold. After trimming, the resulting secondary structures were analyzed to see whether they look like a miRNA hairpin. Further, additional checks were performed so that there were no more than three consecutive mismatches, at least 18–25 nucleotide centered around the miRNA are involved in base-pairing and the hairpin is at least 75 nucleotide in length. Depending on the values set at submission at least 50% of bases in the hairpin should be paired. The most stable valid hairpin from each of the sequence windows was then chosen as the precursor miRNA candidate. After that the precursor miRNA candidate was tested using Randfold (using a cutoff of 0.1).

### Quantitative real time validation of identified miRNAs

For cDNA preparation, 1 μg total RNA (kept equal for each amplification) was subjected to reverse transcription using 20U M-MLV reverse transcriptase (Fermantas, Germany), 1X RT buffer, 20 mM dNTPs (New England Biolabs, USA), 20U RNasin (Fermentas, Germany), 0.1 M DTT with DEPC treated water, and 100 ng of random hexamers (Fermentas, Germany). The expression levels were quantified on ABI7500 Fast system as per manufacturer instructions (Applied Biosystem) using 5 pmol/μl of specific primers with snoRNA142 being taken as endogenous control. Briefly, 20 μl of real time mix contained 10 μl of Power SYBER green master mix (Applied Biosystem), 1 μl cDNA, 6 μl MilliQ water and 1.5 μl of forward, and reverse primers. PCR conditions were set with an initial incubation of 50°C for 2 min, followed by denaturation at 95°C for 10 min, and 40 cycles at 95°C for 15 s, 60°C for 1 min, and 72°C for 40 s. The abundance/ decline of miRNA were normalized to geometric average of endogenous control snoRNA142 for ΔCt. The fold change (ΔCt) was calculated as the difference between infected groups vs. non-infected RAW 264.7 macrophages. The mRNA expression levels were quantified at 24 h post infection.

### Statistical analysis

Statistical analysis of differences between means of groups was determined by two-tailed Student *t*-test and ANOVA on GraphPad Prism 5.0 software. A *p* < 0.05 was considered significant, and a *p* < 0.01 was considered highly significant.

## Results

### *L. donovani* infection in macrophages

To identify altered miRNAs after *L. donovani* infection, we infected macrophages with *L. donovani* (AG83) parasites for 6 h. The intracellular amastigotes in infected macrophages were stained with Diff-Quick (Baxter Healthcare, USA). Approximately 70% macrophages were infected by parasites with a count of 400 ± 250 (mean ± *SD*) parasites per 100 macrophages that were assessed microscopically after 24 h post infection. After 24 h (total 30 h), total RNA was extracted using Tri reagent (Sigma) for further total transcriptome sequencing to identify miRNAs.

### Clustering of differentially regulated miRNAs led us to identify 10 differentially regulated miRNAs in infected macrophages

We describe here a full-scale complete analysis of the miRNA profiles in infected macrophages. A total of 940 miRNAs were identified in parasites infected cells (Supplementary Table 1), out of which 150 miRNAs, shown in heat map (Supplementary Figure 1), were analyzed further. Further, expecting an outsized inter individual unevenness in miRNA expression, we only selected miRNAs that showed consistent tendency of deregulation (either up or down regulated) in the *L. donovani* infection. According to this criterion, only 85 miRNAs (out of 150 miRNAs) had levels consistently modified by *L. donovani* (Supplementary Table 2). On the basis of constant differential expression in parasites infected macrophages, we chose 10 miRNAs (mir-3620, mir-6385, mir-6973a, mir-6996, mir-328, mir-8113, mir-3473f, mir-763, mir-6540, and mir-1264) for their further role in the regulation of macrophage effector functions such as oxidative stress response, phagocytosis, apoptosis, drug response and role in B/T cells lineage commitment, etc.

### The quantitative PCR further confirmed the up and down regulation of identified miRNAs

**Figure 1:**
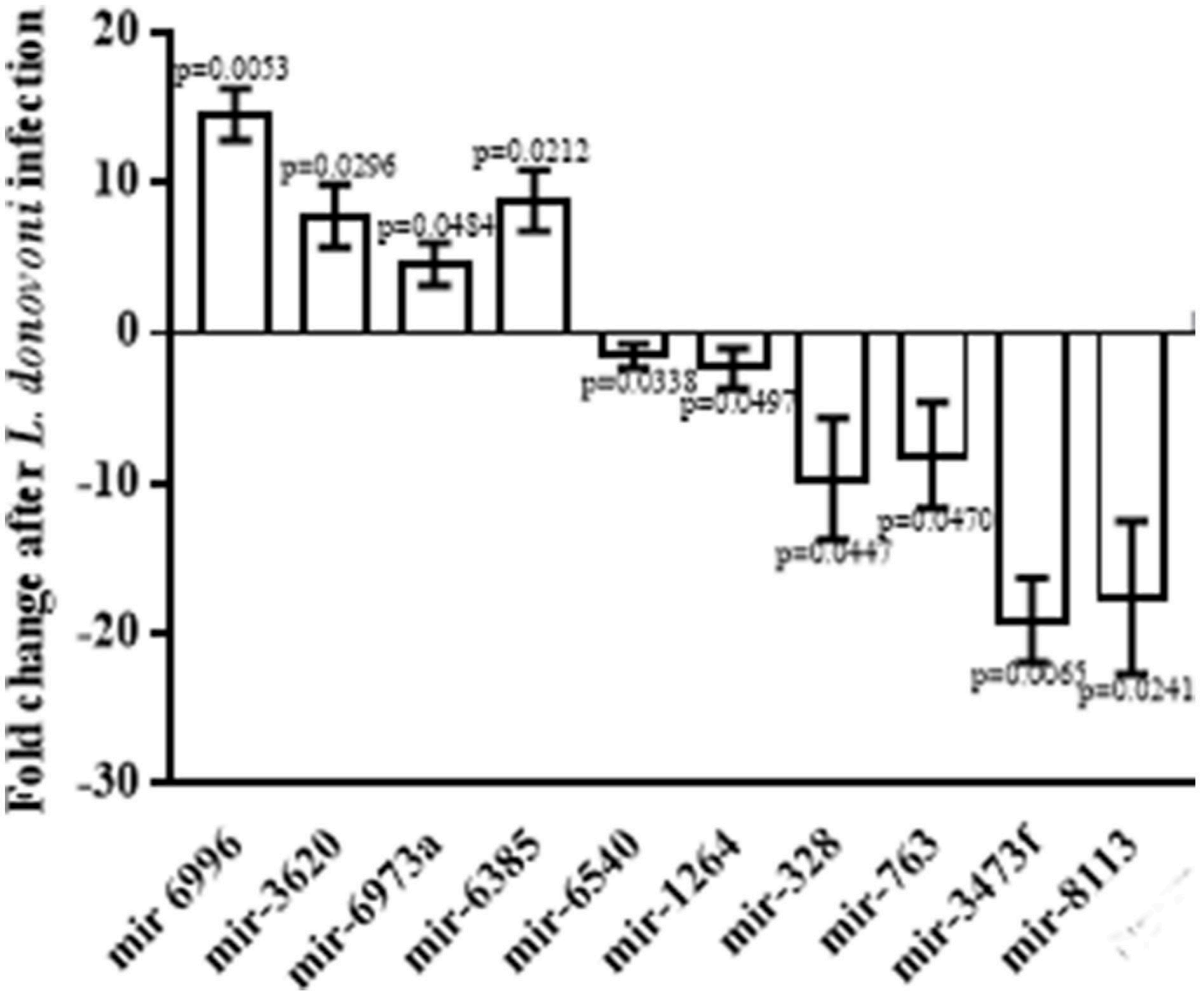
**Relative expression of miRNAs in infected macrophages**. Macrophages were infected with *L. donovani* in a ratio of 1:10 (macrophage: parasite) for 6 h, and then thoroughly washed to remove non-ingested parasites. Macrophages were further incubated at 37°C for 24 h and total RNA was isolated from lysed cells. The miRNAs, mir-6996, mir-3620, mir-6973a, and mir-6385 were found significantly up regulated whereas mir-6540, mir-1264, mir-328, mir-763, mir-3473f, and mir-8113 were found significantly down regulated.

In order to validate the miRNA differential expression data, the quantitative real time PCR (qRT PCR) was done on identified 10 miRNAs. The relative differential expression miRNA was quantified with snoRNA142 that was taken as endogenous miRNA control. Both, the data generated by PCR-array and qRT-PCR confirmed up regulation of mir-6996, mir-3620, mir-6973a, and mir-6385 with fold change 14.56 ± 0.9895 (*p* = 0.0053), 7.823 ± 1.201 (*p* = 0.0296), 4.595 ± 0.8213 (*p* = 0.0484), and 8.831 ± 1.158 (*p* = 0.0212) and down regulation of mir-6540, mir-1264, mir-328, mir-763, mir-3473f, and mir-8113 with fold change −1.505 ± 0.4726 (*p* = 0.0338), −2.318 ± 0.7683 (*p* = 0.0497), −9.688 ± 2.339 (*p* = 0.0447), −8.109 ± 2.047 (*p* = 0.0470), −19.1 ± 1.622 (*p* = 0.0065), and −17.58 ± 2.937 (*p* = 0.0241), respectively as shown in the Figure 1.

### The *de novo* sequencing led us to identify 52 novel miRNAs

The sequencing data after the removal of ligation adapters were analyzed using miRDeep2 tool for the identification of novel miRNAs. A total of 52 novel miRNAs were identified as potential miRNA candidate gene. For each of the novel miRNAs, the corresponding miRNAs precursor and mature sequence were also identified, which confirmed their existence as miRNAs. The chromosome number 10 contains the most number (eight) of novel miRNAs followed by chromosome one which contains six novel miRNAs. There were two novel miRNAs (consensus mature sequence “CUGUACUGUGGAGCCAGC” and “UGGGCAACAGCAGGUCUG”) that were present in more than 800 copy numbers (Supplementary Table 3). These two miRNAs need to be further investigated for their role in leishmanial pathogenesis and susceptibility.

### The miRNA target network validation: identification of miRNA targeted biological processes, molecular functions and cellular components in infected macrophages

For the identification of the transcripts that might be targeted by differentially regulated miRNAs in infected macrophages, we used the validated module of miRBase database to extract miR interaction information. With this approach, several remarkable transcripts virtually targeted by deregulated miRNAs over the infection time course were obtained. The interaction network between miRNAs and miRNA targeted biological processes, molecular functions and cellular components were constructed in Cytoscape 3.2.1 after GO enrichment (Praneenararat et al., 2012).

### The miRNAs were found to regulate various biological processes linked to macrophage dysfunction and survival in parasite

**Figure 2:**
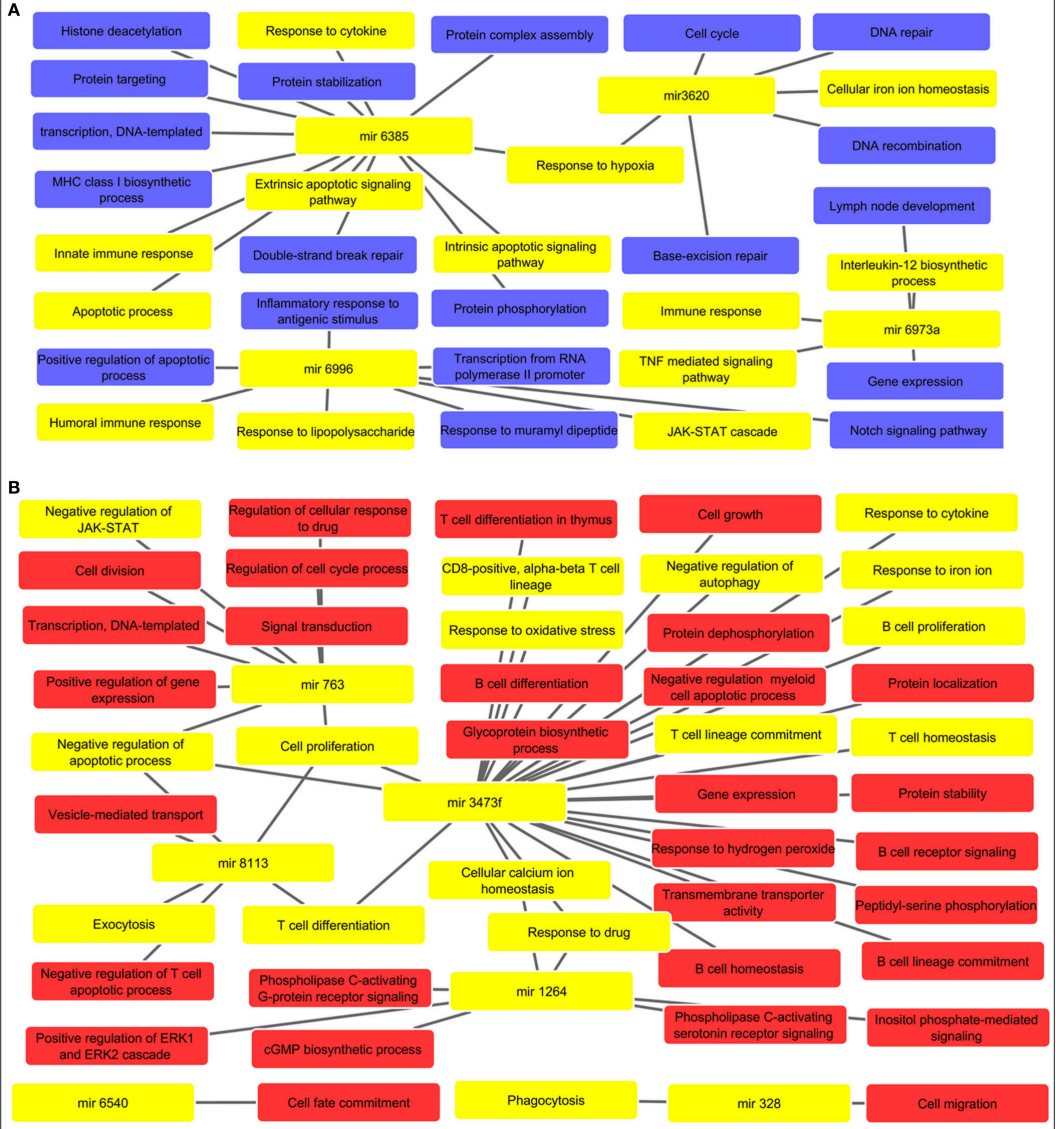
**(A)** Predicted biological processes of up regulated miRNAs after GO enrichment. The interlinked miRNAs and important pathways linked to macrophages effector functions such as phagocytosis, ROS metabolic process, B/T cell differentiation, etc. during pathogenic invasion are highlighted with yellow color. **(B)** The interaction of down regulated miRNAs and their targeted biological processes. The highlighted yellow color denotes interlinked miRNAs with key biological processes relevant to macrophage dysfunction such as cytokine response, immune response, negative regulation of apoptotic process, response to oxidative stress, etc.

Biological processes were inferred from analysis of both, up and down regulated miRNA targets, which are depicted in Figures 2A,B, respectively. The list of miRNAs linked pathways is given in Supplementary Tables 4, 5 for up and down regulated pathways, respectively. The GO enrichment identified the involvement of these miRNAs in various biological processes such as cell cycle (mir-3620), cytokine response (mir-3473f and mir-6385), cell proliferation (mir-3473f and mir-8113), response to LPS (mir-6996), Th1/Th2 phenotype dichotomy (mir-3473f and mir-8113), phagocytosis (mir-328), protein complex assembly and it's stabilization (mir-6385), ROS metabolism (mir-3473f), response toward oxidative stress (mir-3473f), apoptosis (mir-3473f, mir-8113, and mir-6385), immune response (mir-6996 and mir-6385), and JAK-STAT signaling pathway (mir-763 and mir-6996). These all biological processes are relevant not only for host cells but also to counter attack intracellular parasites.

### The identified miRNAs were found to be associated with important molecular functions that may result in dampened macrophage effector functions

**Figure 3:**
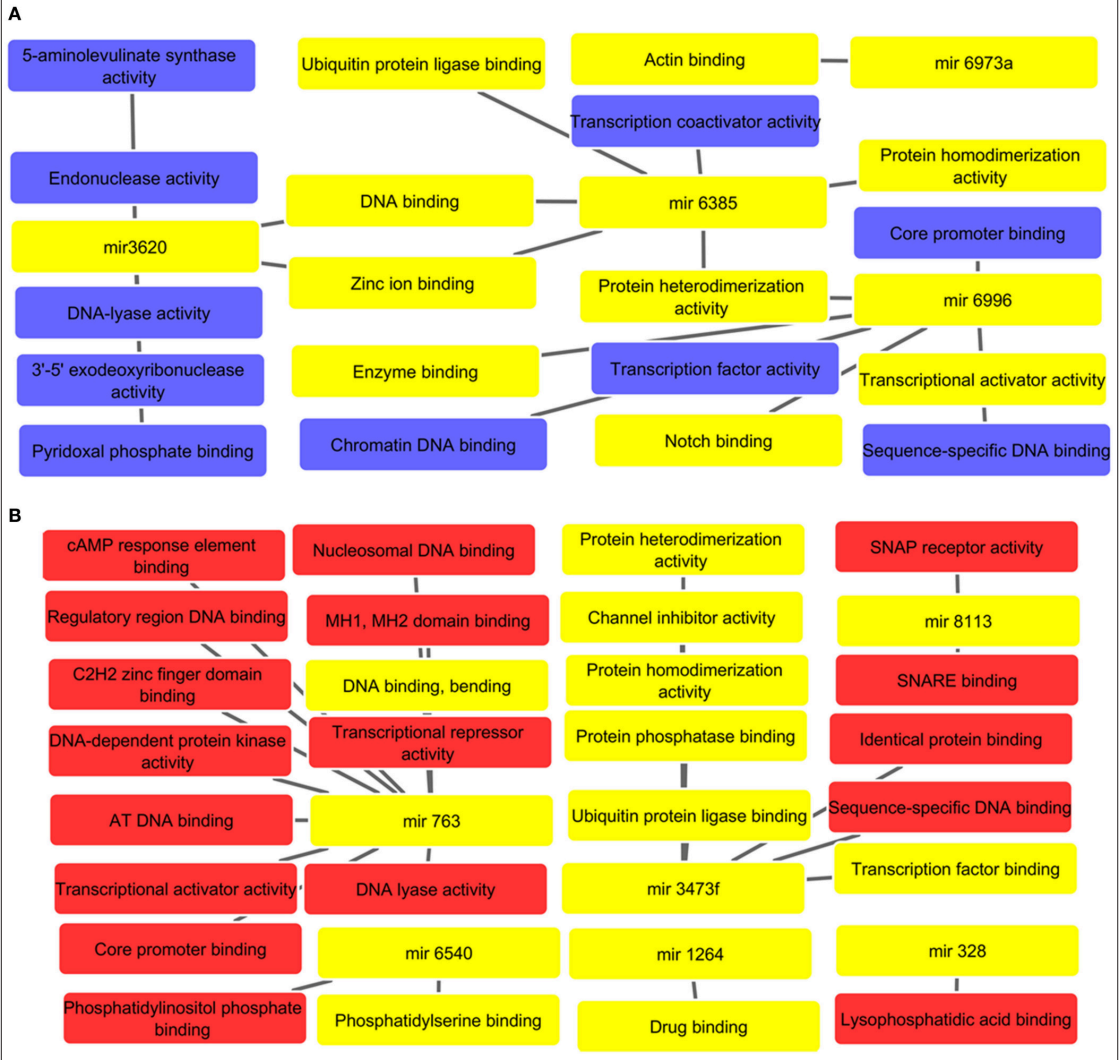
**(A)** Molecular network of up regulated miRNAs. All interlinked miRNAs and their respective targets have been highlighted with yellow color. The important molecular functions linked to cellular dysfunction such as DNA, enzyme and actin binding are highlighted with yellow color. **(B)** Analysis of molecular functions of down regulated miRNAs in *L. donovani* infected RAW 264.7 macrophages at 24 h post-infection with highlighted yellow color denoting important molecular functions such as drug binding, phosphatidylserine binding, protease, and transcription factor binding, etc.

The potential molecular functions linked targets for up and down regulated miRNAs are represented in network diagram as depicted in Figures 3A,B, respectively. All supporting and detailed information are clubbed in Supplementary Tables 6, 7, respectively. The GO enrichment studies revealed association with protein dimerization (mir-6385), transcription activator activity (mir-6996), and regulation of zinc ion binding (mir-6385 and mir-3620). The down regulated miRNAs, were found to regulate DNA, core promoter, phosphatidylserine binding (mir-6540) binding, transcription co-activation, and protein homo-dimerization activities (mir-3473f, mir-763, and mir-6385), and drug binding pathways (mir-1264), etc. These all molecular functions especially phosphatidylserine and drug binding are vital to restrict intracellular parasites survival. Hence, it is with great value to decipher how alterations of these miRNAs can result in macrophage dysfunction in leishmanial infection.

### Cytoscape interaction analysis identified the involvement of miRNAs in various cellular components required for cellular functions and integrity

**Figure 4:**
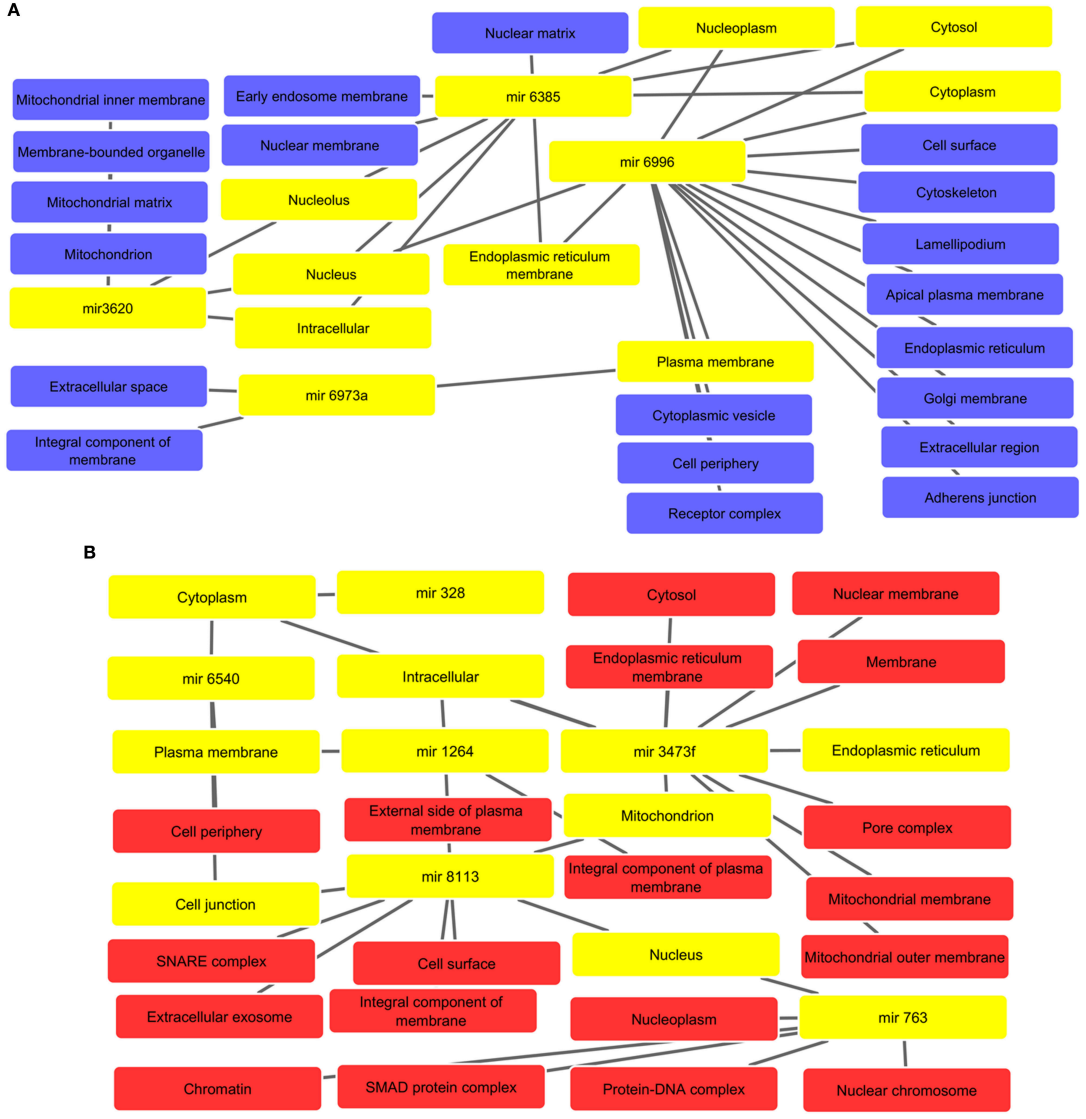
**(A)** Associated cellular components of up regulated miRNAs with interlinked miRNAs and their respective key cellular compartments highlighted with yellow color such as nucleoplasm, cytoplasm, and intracellular, etc. **(B)** Cellular components inferred from analysis of down regulated miRNA-targets. The highlighted yellow color denotes interlinked miRNAs and their respective key cellular components like cytoplasm, plasma membrane, mitochondrion, and cell junction, etc.

The interaction analysis revealed that altered miRNAs are located in the various components such as nuclear, mitochondrial, and plasma membranes, cytoplasm, endoplasmic reticulum, golgi membrane, and cytoskeleton, etc. The interactive networks for up and down regulated miRNAs are depicted in Figures 4A,B. All supported information is included in Supplementary Tables 8, 9, respectively.

## Discussion

Notwithstanding, we have made considerable progress in leishmanial diagnostic and pathology however; the parameters of diseases susceptibility and protective immunity are not well identified. How does *Leishmania* prime host macrophages in its favor? It is still a matter of investigation. In this study, we identified 150 miRNAs using *de novo* sequencing approach, which expression levels were largely altered in *L. donovani* infected macrophages. Further, the *in silico* analysis acknowledged the important of role of 10 miRNAs that possibly facilitate parasite survival through down regulation of macrophage effector genes. In addition, this approach led us to identify two novel miRNAs that were present in huge copy numbers (>800), which suggested that *Leishmania* significantly alters host miRNAs machinery in its favor at early stage of infection.

*Leishmania* species are digenetic obligatory intracellular parasitic protozoans that reside inside two hostile environments i.e., the midgut of the insect vector, and the phagolysosomes of the mammalian macrophages (Sadlova et al., 2016). The initial interaction between the host cell and parasite determines whether parasite will make silent entry in macrophages and persist or host will eliminate the parasite (Stager et al., 2010). Since the identified miRNAs were altered soon after infection i.e., 30 h post infection, it seems that *Leishmania* significantly inhibits these biological processes through invention in gene regulatory mechanisms. During the initial phase, a variety of host cell receptors (CR1, CR3) and parasite molecules (LPG, gp63) facilitate entry of parasites in host macrophages albeit the exact mechanisms are not well understood. It is established that *Leishmania* expresses phosphatidylserine (PS) in the outer leaflet of cytoplasmic membrane, which acts as a signal for the macrophages and enables silent invasion (Liu and Uzonna, 2012). It is also believed that the parasite expresses equal proportion of phosphatidylserine on dead parasites as compared to viable parasites. The dead parasites are engulfed by the macrophages silently without activating them as they are supposed to be harmless. This enables the normal viable parasites to enter silently within the macrophages either at the same time when phosphatidylserine expressing dead parasites are phagocytized or later. We identified a miRNA, mir-6540 that was linked to phosphatidylserine binding and significantly down regulated therefore; further studies might be handy to reveal silent entry of *Leishmania* in host macrophages.

*Leishmania* promastigotes are highly susceptible in blood stream of infected host therefore, they ligate host cell receptors and trigger phagocytosis for internalization to increase their chances of survival soon after the infection (Ueno and Wilson, 2012; Gupta et al., 2013). The parasites acquire nutrients especially cations for its transformation and replication in the host phagolysosomes. The regulation of phagocytosis and phagocytic vesicle formation was found associated with the biological processes affected by mir-328, which was down regulated in infected cells that further offers a candidate for investigation. Further, after phagocytosis parasite requires little time for its growth and replication; therefore, it is quite likely that it delays host cell apoptosis along with down regulating macrophages effector properties (Moore and Matlashewski, 1994). The mir-3473f, mir-763, and mir-8113 were found to be associated with negative regulation of apoptotic process, which further suggested the possibility of these miRNAs to obstruct normal functions of macrophage activation.

*Leishmania* possess lipophosphoglycan2 (LPG2) and leishmanolysin (gp63), which are well known modulators of host immune response via regulation of its complement and phagocytosis process along with nitric oxide-mediated signal transduction (Olivier et al., 2005). Therefore, their association with host miRNAs expression may reveal its survival strategies in host macrophages. We found a strong association of mir-6996 toward LPS thus it is likely that this miRNA might be involved in LPG and gp63 related signaling and dominance on host macrophages as its expression in infected cells was significantly up regulated.

In visceral Leishmaniasis, the host ability of parasite resistance is characterized by generation of Th1 response, which produce pro-inflammatory cytokines such as IL-2, IL-12, etc. whereas generation of Th2 response, characterized by IL-4 and TGF-β secretion, favors for survival of the parasite inside the host (Singh et al., 2012; Rodrigues et al., 2014). So far, the host and parasitic factors responsive for the Th1 to Th2 paradigm shift are not identified. We found that mir-3473f and mir-8113 might be the possible candidates to regulate T cell proliferation, differentiation, and Th1/Th2 dichotomy in leishmanial pathogenesis as these were found down regulated, and hence requires further studies. Further, the highly induced mir-6973a was found to be associated with IL-12 biosynthesis, a key molecule in regulating cellular immune system and essential for activating Th1 responses. Therefore, through up-regulation of this miRNA, parasite might be able to prevent IL-12 production and shift protective Th2 type response to Th2 type for its continual survival and proliferation. Therefore, these miRNAs may be further investigated for their role in *Leishmania* induced Th2 type immune response and Th1 to Th2 phenotype change. The mir-3473f was also found to be linked with autophagy inhibition, which further suggests its possible role in leishmanial pathogenesis as autophagy keeps a check on intracellular pathogens (Frank et al., 2015).

*Leishmania* acquires host divalent cations especially iron for its cellular division and proliferation and also for peroxidases, the major defense enzymes in host macrophages (Singh et al., 2013). Iron homeostasis is an important host strategy to export iron from macrophage cytoplasm to ensure its least availability for intracellular parasites. The mir-3620 was found to be linked to cellular iron homeostasis, and since it was significantly elevated, possibly it down regulates genes of iron homeostasis to ensure maximum availability of iron in cell cytoplasm. In addition, along with mir-3620, the mir-6385 was also found to regulate hypoxia. Hypoxia induces macrophages to control leishmanial infections (Degrossoli et al., 2011) since these miRNAs were highly elevated that probably help to down regulate hyoxia inducing genes and hence require more studies.

MiRNAs have also shown to play important role in drug resistance in both, infectious and non-infectious diseases (To, 2013; Ren et al., 2015). Studies also suggest the role of *ldmdr1* and ATP binding cassette (ABC) proteins in disease resistance and susceptibility in leishmanial pathogenesis (Henderson et al., 1992; Singh et al., 2014). However, the abnornal expression of these has not been correlated with miRNAs regulation so far. The GO enrichment revealed that mir-763, mir-1264, and mir-3473f also regulate drug response mechanisms. Thus it is possible that *Leishmania* induces the macrophages to over express such proteins (for e.g., ABC transporters) by down regulating these miRNAs, which help to efflux out drugs from the cell.

## Conclusion

To conclude, we identified a total of 940 miRNAs by *de novo* sequencing in *L. donovani* infected macrophages. A combination of 150 miRNAs, which were widely altered in infected macrophages of which 10 miRNAs were found to play significant role in the regulation of macrophage effector functions. These findings emphasize the potential of miRNAs in regulation of macrophage effector functions and highlight their importance in leishmanial pathogenesis that can be targeted to curb visceral leishmaniasis. In addition, the identified miRNAs could also be used as biomarkers to distinguish various pathological states such as cured, symptomatic and asymptomatic VL infections, and also can be used to design new drug targets and control strategies.

## Author contributions

NT, VK, MG, and AS: designed and performed the experiments and co-wrote the MS. SS and VS: assisted in design of the study, statistical analysis, and MS writing. RS: conceived, designed, directed, and supervised the complete study.

### Conflict of interest statement

The authors declare that the research was conducted in the absence of any commercial or financial relationships that could be construed as a potential conflict of interest. The reviewer HM and handling Editor declared their shared affiliation and the handling Editor states that the process nevertheless met the standards of a fair and objective review.

## Supplementary material

Supplementary Figure 1 **miRNA expression signature in *L. donovani* infected and non-infected macrophages**. The miRNA expression values are presented using a green-black-red color scheme. Clustering analysis of 150 highly expressed miRNAs are shown in heat map. Expecting a large inter individual variability in miRNA expression, we only selected miRNAs that showed consistent trends of deregulation (either up- or down-regulated) in the RAW 264.7 mice macrophages infected with *L. donovani* and only 85 miRNAs had levels consistently modified by parasite infection.

Supplementary Table 1 **Differential miRNA expression (ranked from most differentially expressed to least) levels of 940 macrophage miRNAs in *L.donovani* infected RAW 264.7 macrophages**. Results were obtained at 24 h post infection(.xlsx).

Supplementary Table 2 **Pooling of differential expression of 150 miRNAs**. Expecting a large inter individual variability in miRNA expression, in the RAW 264.7 mice macrophages infected with *L. donovani* and only 85 miRNAs were selected having levels consistently modified by parasite infection (.xlsx).

Supplementary Table 3 **Identified novel miRNAs in *Leishmania* infected macrophages**.

Supplementary Table 4 **Analysis of up regulated miRNAs (mir-6996, mir-3620, mir-6973a, and mir-6385) and their targeted biological processes targets in *L. donovani* infected RAW 264.7 macrophages**. Tables indicate the miRNA name, GO id, and function (.xlsx).

Supplementary Table 5 **Biological processes inferred from analysis of down regulated miRNA-targets (mir-6540, mir-1264, mir-328, mir-763, mir-3473f, and mir-8113) in *L. donovani* infected RAW 264.7 macrophages**. Tables indicate the miRNA name, GO id, and function (.xlsx).

Supplementary Table 6 **Molecular functions of up regulated miRNA-targets (mir-6996, mir-3620, and mir-6385) in *L. donovani* infected RAW 264.7 macrophages**. Tables indicate the miRNA name, GO id, and function (xlsx).

Supplementary Table 7 **Molecular functions inferred from analysis of down regulated miRNA-targets (mir-6540, mir-1264, mir-328, mir-763, mir-3473f, and mir-8113) in *L. donovani* infected RAW 264.7 macrophages**. Tables indicate the miRNA name, GO id, and function (.xlsx).

Supplementary Table 8 **Up regulated miRNA-targets (mir-6996, mir-3620, mir-6973a, and mir-6385) and their respective targeted cellular components in *L. donovani* infected RAW 264.7 macrophages**. Tables indicate the miRNA name, GO id, and Cellular components (.xlsx).

Supplementary Table 9 **Cellular components inferred from analysis of down regulated miRNA-targets (mir-6540, mir-1264, mir-328, mir-763, mir-3473f, and mir-8113) in *L. donovani* infected RAW 264.7 macrophages**. Tables indicate the miRNA name, GO id, and Cellular components (.xlsx).
