# Corrigendum: Identification and Characterization of miRNAs in Response to *Leishmania donovani* Infection: Delineation of Their Roles in Macrophage Dysfunction

**DOI:** 10.3389/fmicb.2017.01190

**Published:** 2017-06-27

**Authors:** Neeraj Tiwari, Vinod Kumar, Mallikarjuna Rao Gedda, Ashish K. Singh, Vijay K. Singh, Surya P. Singh, Rakesh K. Singh

**Affiliations:** ^1^Molecular Immunology Group, Department of Biochemistry, Institute of Science, Banaras Hindu UniversityVaranasi, India; ^2^Department of Parasitology and Molecular Biology, Rajendra Memorial Research InstitutePatna, India; ^3^Bioinformatics Programme, Centre for Biological Science, Central University of South BiharPatna, India

**Keywords:** *L. donovani*, miRNA, biomarkers, macrophages, dysfunction

It has come to our attention that Dr. Sreenivas Gannavaram does not meet criteria necessary to be listed as an author, as specified by Frontiers; therefore, he has been removed from the authors' list and mentioned Acknowledgments instead.

The updated Author Contributions statement and Acknowledgments are below.

The authors apologize for this error and state that it does not change the scientific conclusions of the article in any way.

The original article has been updated.

## Author contributions

NT, VK, MG, and AS: designed and performed the experiments and co-wrote the MS. SS and VS: assisted in design of the study, statistical analysis, and MS writing. RS: conceived, designed, directed, and supervised the complete study.

### Conflict of interest statement

The authors declare that the research was conducted in the absence of any commercial or financial relationships that could be construed as a potential conflict of interest.

