# Supplementary figures and images for: Identification and Characterization of miRNAs in Response to Leishmania donovani Infection: Delineation of Their Roles in Macrophage Dysfunction
Source: Front Microbiol. 2017 Mar 2;8:314. doi: 10.3389/fmicb.2017.00314 (PMC5332369; doi:10.3389/fmicb.2017.00314)

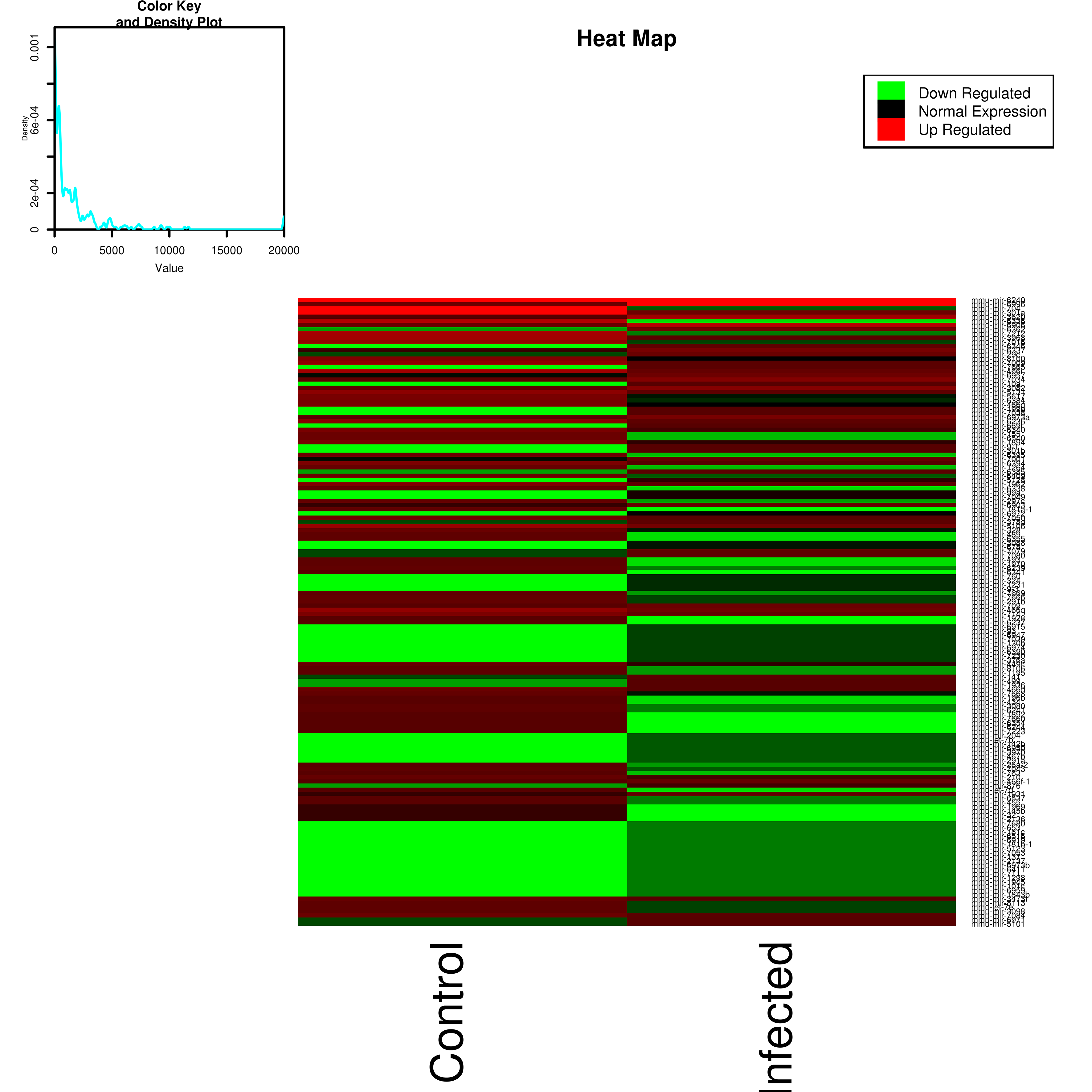

Supplement: Supplementary Figure 1 — miRNA expression signature in L. donovani infected and non-infected macrophages. The miRNA expression values are presented using a green-black-red color scheme. Clustering analysis of 150 highly expressed miRNAs are shown in heat map. Expecting a large inter individual variability in miRNA expression, we only selected miRNAs that showed consistent trends of deregulation (either up- or down-regulated) in the RAW 264.7 mice macrophages infected with L. donovani and only 85 miRNAs had levels consistently modified by parasite infection. [file Image1.TIF]
